# COVID-19 Testing Trend: A Retrospective Analysis of the Three Major Pandemic Waves in Punjab, Pakistan

**DOI:** 10.7759/cureus.52309

**Published:** 2024-01-15

**Authors:** Rabia M Chaudhry, Sadia Minhas, Mehroz A Khan, Shumaila Nargus, Kanza Nawadat, Muhammad Athar Khan, Muhammad Kashif

**Affiliations:** 1 Oral Medicine, Akhtar Saeed Medical and Dental College, Lahore, PAK; 2 Public Health, University Institute of Public Health, The University of Lahore, Lahore, PAK; 3 Microbiology, The University of Lahore, Lahore, PAK; 4 Oral Pathology, Akhtar Saeed Medical and Dental College, Lahore, PAK; 5 College of Dentistry, Akhtar Saeed Medical and Dental College, Lahore, PAK; 6 Oral and Maxillofacial Surgery, Bakhtawar Amin Medical and Dental College, Multan, PAK; 7 Oral Pathology, Bakhtawar Amin Medical and Dental College, Multan, PAK

**Keywords:** pakistan, lower middle income countries, pandemic, testing trend, covid-19, infectious disease

## Abstract

Background/objectives: There is some evidence in the literature of under-testing of COVID-19 cases in Pakistan. This study aims to explore COVID-19 testing trends and the factors affecting them in a lower middle-income country for future infectious disease policy-making and intervention strategies.

Methodology: The study was conducted as a serial cross-sectional study during the three major peaks from March 2020 to June 2021 on 1616 participants in Punjab, Pakistan. This is the first study to explore COVID-19 testing trends in association with flu-like symptoms (FLS) and the factors affecting all three major waves in Pakistan.

Results: The results show that in all three waves, only 18.8% reported COVID-19 tested despite that 86.7% thought they had already had COVID-19, with 51.3% reporting having FLS and 35.6% with exposure to FLS from their families and 19.8% of positive testing rate among their family members. Out of the survey participants, 66% received vaccination, and over 80% had their eligible family members immunized. Fear of contracting COVID-19 was 69.7% in all three waves. Factors positively associated with the uptake of testing were the age group of 31-40 years with an adjusted odds ratio of 3.27 (95% confidence interval (CI): 2.09-5.12) for the second wave and an adjusted odds ratio of 13.75 (95% CI: 9.43-20.01) for the third wave and traveling abroad with odds of 3.08 times when the reference was inland traveling. The adjusted odds ratio to test for FLS was 1.62 (95% CI: 1.21-2.16).

Conclusion: In this study, there is convincing evidence of COVID-19 under-testing and thus under-reporting. This study also suggests that fear-based interventions may be counterproductive; however, economic factors such as education, employment, and traveling are significant in guiding the behavior for infectious disease prevention and management.

## Introduction

In recent years, the world has seen one of the largest pandemics in history. This outbreak of the deadly disease was caused by a novel coronavirus known as severe acute respiratory syndrome coronavirus 2 (SARS-CoV-2) also known as coronavirus disease of 2019 (COVID-19). It was first officially reported in December 2019, in the district of Wuhan. Since then, this virus spread around in approximately 215 countries [1]. The World Health Organization (WHO) declared it a global pandemic on 11 March 2020 and at the time, a total of 17 million cases were reported with 6896035 deaths worldwide. Pakistan reported its first case of COVID-19 on 26 February 2020, with 1581211 confirmed cases and 30661 deaths as of 3 July 2023, which constitutes about 0.019% of the total cases worldwide.

COVID-19 spreads between humans through respiratory droplets of asymptomatic and symptomatic patients (fever, cough, fatigue, and dyspnea) [2,3]. Furthermore, there is confirmation that COVID-19 risk factors are improperly distributed geographically, with a raised tendency to cluster in areas defined by socioeconomic, racial, and rural characteristics [4]. This might result in COVID-19 testing disparities, raised incidence of disease, and unfavorable health outcomes in a few areas in contrast to others [5,6].

The government of Pakistan implemented many strategies to overcome this pandemic. A country-wide lockdown was implemented, and strict testing was done. Pakistan initiated a smart, partial lockdown policy in which only the most affected regions of the country were put under lockdown [1,7]. This strategy was lauded by many international strategists and then many countries implemented it. Along with these lockdowns, testing facilities were also established throughout the country. At the start of the pandemic, testing was not common, and the suspected cases were sent to foreign laboratories. Later, throughout the country, laboratories were equipped with a COVID-19 PCR testing facility. The WHO also set test centers for COVID-19 in seven hospitals [8]. The testing capacity was raised from 30,000 to 280,000 and further increased to 900,000. The National Disaster Management Authority (NDMA) worked with the National Institute of Health (NIH) to increase the number of coronavirus testing laboratories from 15 to 50 [1,7,8]. The Pakistan Ministry of National Health Services (NHS) encouraged testing those with mild and moderate symptoms. Furthermore, they categorized the individuals into high-priority and priority individuals. Within Pakistan, all tests reported by the NHS used the polymerase chain reaction (PCR) test to confirm COVID-19 infection [7].

Despite all the efforts, from many areas of Pakistan, there were reports of under-testing of COVID-19. This low trend of testing was mainly attributed to the limited testing capacity of the country and thus the plausibility of more COVID-19 cases than reported [9]. However, to the best of our knowledge, there is no study yet from Pakistan that explores the role of people’s attitude in this trend of COVID-19 under-testing in the country and the factors affecting it. Thus, this study is designed to study the Pakistani population’s role in COVID-19 testing disparities and the associated encouraging and discouraging factors. This is also the first study to determine the association of COVID-19 testing with FLS and the factors affecting all three major COVID-19 peaks in Pakistan.

## Materials and methods

Study design

The study was a serial cross-sectional analytical study conducted in Punjab, the most populous province of Pakistan during the three waves of COVID-19 from March 2020 to September 2021 with a total of 1616 participants. The initial set of data was collected between March 2020 and August 2020, followed by a subsequent set from September 2020 to January 2021, and a final set from February 2021 to July 2021.

Study population

The study population was the general Pakistani population of Punjab. The inclusion criteria were being aged > 17 years, both genders, residents of Punjab, and willingness to be a part of the survey. Surveys with incomplete data were excluded from the study. Electronic informed consent was obtained from all participants before they participated in the survey. The confidentiality of participants was ensured by keeping the data anonymous and secure.

Data collection

A web-based convenient sampling technique was used for the collection of data. A 23-item structured questionnaire was constructed in English and Urdu for the study based on available WHO-published data on COVID-19 at the time. The research team for this study calculated questionnaire content validity by a subjective approach. The research team subjectively evaluated the language, relevance, and presentation of questions [10]. The questionnaire encompassed information regarding participants' socio-demographics, travel records, flu-like symptoms (FLU), and COVID-19 testing, including test results and vaccination status. The collected responses were saved in CSV files for subsequent analysis.

Study variables

Outcome Variable

The outcome variable in this study was testing for COVID-19. Participants were asked whether they had been tested for COVID-19 and the results of the test (positive, negative, or unknown). The primary aim of the study was to examine the predictors of COVID-19 testing during the three waves of the pandemic in Pakistan.

Explanatory Variables

Participants were categorized into three groups based on the wave of COVID-19 during which they completed the survey (first wave, second wave, or third wave). Participants were asked to report their age in years. Age was treated as a continuous variable in the analysis. Participants were asked to report their gender as male or female. Participants were asked whether they had traveled abroad or within the country during the pandemic. This variable was categorized as "abroad" or "inland". Participants were asked whether they had experienced any FLU (such as fever, cough, sore throat, or body aches) in the past 14 days. Participants were asked if they were exposed to FLS through their families.

Ethical consideration

The confidentiality of participants was ensured by keeping the data anonymous and secure. The study was performed following the Declaration of Helsinki as revised in 2013. The study was conducted following the Checklist for Reporting Results of Internet E-Surveys (CHERRIES) guidelines. This study was approved and reviewed by the Akhtar Saeed Medical and Dental College Institutional Review Board (IRB).

Statistical analysis

IBM SPSS Statistics for Windows, Version 20 (Released 2011; IBM Corp., Armonk, New York, United States) was used to perform all statistical analyses in this study. Descriptive statistics were used to summarize the characteristics of the study sample. Frequencies and percentages were used to describe the data for each wave of COVID-19, as well as for the categorical explanatory variables (gender, traveling history, FLS, and test results). For analytical statistical analysis, the Chi-square test was used to compare the distribution of categorical variables (gender, traveling history, FLS, exposure, and test results) across different waves of COVID-19. The Fisher's exact test was used when the cell counts for a variable were small (less than 5) to determine whether there was a significant difference in the distribution of categorical variables across different waves of COVID-19. The likelihood ratio test was used to compare the goodness of fit between various nested models, the model with the best fit, and finally, binary logistic regression analysis was used to examine the relationship between the outcome variable (COVID-19 testing) and the explanatory variables (waves of COVID-19, age, gender, traveling history, and FLS) while controlling for potential confounding factors. Adjusted odds ratios with 95% confidence intervals were used to report the results. The statistical significance level was set at p ≤ 0.05.

## Results

In this study, a total of 1,616 individuals responded to the survey during three waves of the COVID-19 outbreak in Pakistan. Of these, 782 people responded in the first wave, while 359 and 475 responded in the second and third waves, respectively. The majority of the respondents (60.9%) were between the ages of 21 and 30, followed by those aged 17-20 years. The females comprised 69.6% of the study participants (Table 1).

**Table 1:** Basic demographic characteristics of respondents by pandemic waves.

		Time								p-value
		First wave (Feb 2020) (n = 782)		Second Wave (Aug 2020) (n = 359)		Third wave (May 2021) (n = 475)		Total (n = 1616)		
		N	%	n	%	n	%	n	%	
Age	10-20	228	29.2	51	14.2	122	25.7	401	24.8	<0.001
	21-30	496	63.4	202	56.3	286	60.2	984	60.9	
	31-40	38	4.9	66	18.4	18	3.8	122	7.5	
	41-50	4	0.5	20	5.6	27	5.7	51	3.2	
	51-60	6	0.8	9	2.5	15	3.2	30	1.9	
	61-70	6	0.8	10	2.8	4	0.8	20	1.2	
	71-80	4	0.5	1	0.3	3	0.6	8	0.5	
Gender	Male	217	27.7	125	34.8	150	31.6	492	30.4	0.045
	Female	565	72.3	234	65.2	325	68.4	1124	69.6	

**Figure 1:**
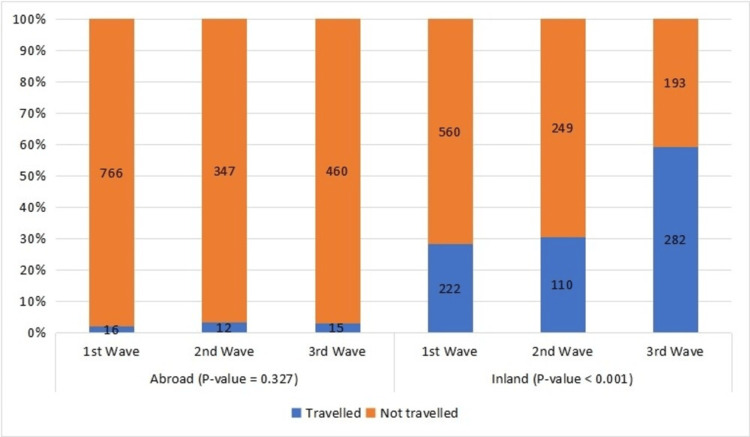
Traveling history of the participants in the three pandemic waves.

During the first wave, only 2% of the respondents had a travel history abroad and 28.4% had a travel history within the country, while during the third wave, 59.4% of the respondents reported traveling within the country (Figure 1, Table 1).

Table 2 shows that most of the participants (90.1%) reported having no chronic conditions, while 9.2% reported having only one chronic disease, and 0.7% reported having two chronic diseases. Diabetes, hypertension, asthma, and allergies were the most reported chronic conditions. None of the other chronic conditions were reported in more than 10 cases overall during the three waves (Table 2).

**Table 2:** Comorbidities of the participants by pandemic waves.

No. of comorbid conditions	Time								
	First wave (Feb 2020) (n = 782)		Second Wave (Aug 2020) (n = 359)		Third wave (May 2021) (n = 475)		Total (n = 1616)		
	n	%	n	%	N	%	n	%	
No	701	89.6	329	91.6	425	89.5	1455	90.1	
Single	75	9.6	28	7.8	46	9.7	149	9.2	
Two	6	0.8	2	0.6	4	0.8	12	0.7	
Total	782	100.0	359	100.0	475	100.0	1616	100.0	

The FLS were most prevalent during the first wave of COVID-19 in Pakistan, with 61.5% of respondents reporting symptoms. FLS were least prevalent during the second wave (29.2%) and then increased again during the third wave (51.2%). The difference in FLS prevalence across the three waves was found to be statistically significant (p < 0.001). Additionally, the proportion of respondents who suspected they had COVID-19 was found to be significantly higher during the third wave compared to the first and second waves (p < 0.001) (Table 3).

**Table 3:** FLS prevalence, COVID-19 testing, and positivity rate. FLS: Flu-like symptoms

Personal Symptoms, Test, and Result		Time								p-value
		First wave (Feb 2020) (n = 782)		Second Wave (Aug 2020) (n = 359)		Third wave (May 2021) (n = 475)		Total (n = 1616)		
		n	%	n	%	n	%	n	%	
Have you had any flu-like symptoms since Feb/September 2020?	No	301	38.5	254	70.8	232	48.8	787	48.7	<0.001
	Yes	481	61.5	105	29.2	243	51.2	829	51.3	
Do you think you have had COVID-19?	No	712	91.0	332	92.5	357	75.2	1401	86.7	<0.001
	Yes	70	9.0	27	7.5	118	24.8	215	13.3	
Have you been tested for coronavirus?	No	739	94.5	304	84.7	270	56.8	1313	81.2	<0.001
	Yes	43	5.5	55	15.3	205	43.2	303	18.8	
If yes, what is the result?	Negative	26	60.5	38	69.1	133	64.9	197	65.0	0.672
	Positive	17	39.5	17	30.9	72	35.1	106	35.0	

**Figure 2:**
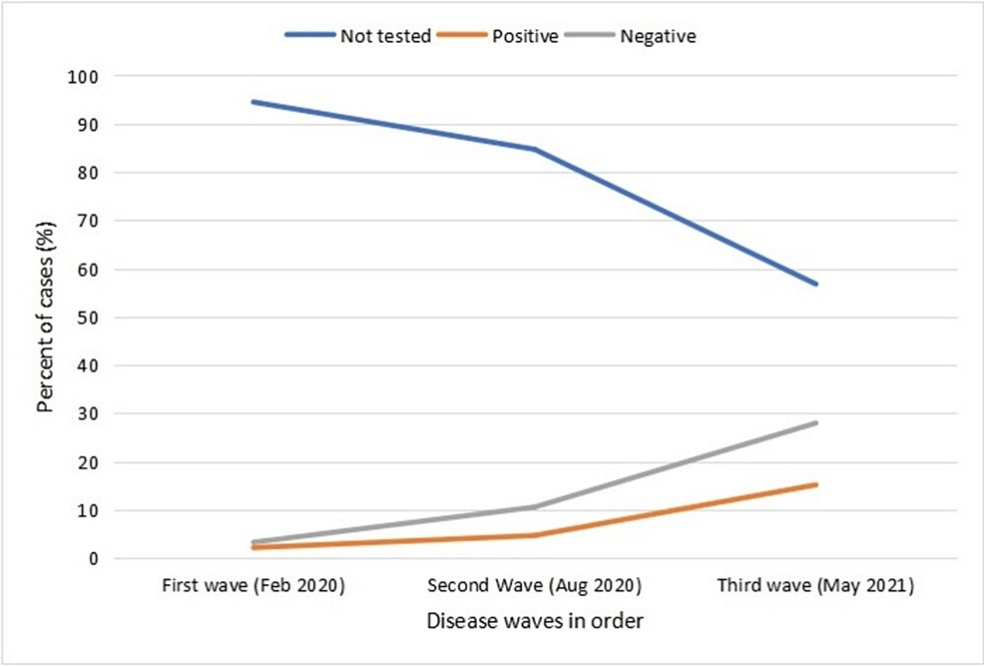
Line graph presenting trends of not tested, positive, and negative cases.

The study found that the overall testing done among the study participants was n = 303 (18.8%) with an increasing trend over time (first wave = 5.5%, second wave = 15.3%, third wave = 43.2%). The overall positivity rate among the tested participants was 35% which was found to be highest during the first wave (39.5%) and lowest during the second wave (30.9%), with a slight increase again during the third wave (35.1%) (Table 3, Figure 2).

**Figure 3:**
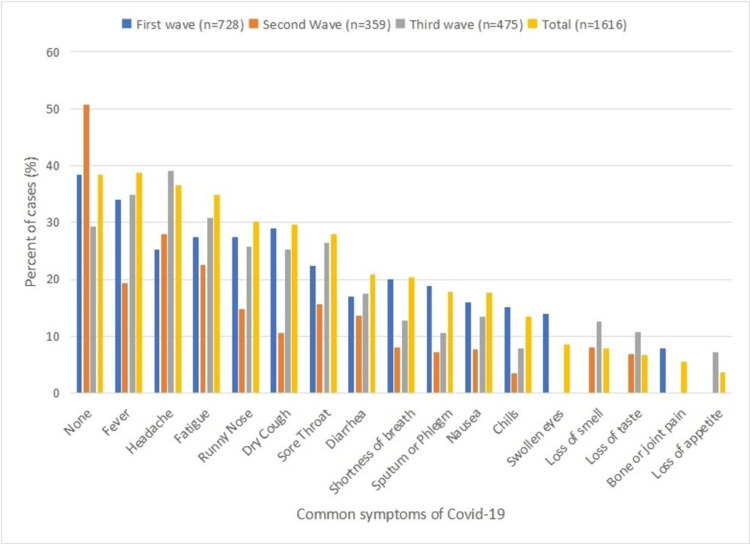
Symptoms in order of presence among total cases.

Fever was the most common symptom reported across all three waves of COVID-19 in Pakistan, while other symptoms (headache, runny nose, dry cough, sore throat, and diarrhea) were more prevalent during the first and third waves compared to the second wave. Shortness of breath, sputum or phlegm, nausea, and chills were more common during the first wave, reduced during the second wave, and then slightly increased again during the third wave (Figure 3, Table 3).

The study reported that among their family members, the FLS were more prevalent during the third wave in contrast to the first wave and second wave (54.7% vs. 27.9% vs 27.3%). The overall positivity rate for COVID-19 infection among respondent family members, 19.8%, was significantly higher during the third wave (25.3%) compared to the first and second wave (17.3 vs 15.9%) (Table 4).

**Table 4:** FLS and COVID-19 testing of respondents' family members by pandemic waves. FLS: Flu-like symptoms

Family symptoms, test, and results		Time								p-value
		First wave (Feb 2020) (n = 782)		Second Wave (Aug 2020) (n = 359)		Third wave (May 2021) (n = 475)		Total (n = 1616)		
		n	%	n	%	n	%	n	%	
Do you think any of your family members has had one of the symptoms mentioned in Q.7	No	564	72.1	261	72.7	215	45.3	1040	64.4	<0.001
	Yes	218	27.9	98	27.3	260	54.7	576	35.6	
Did you have a family member who got tested positive for Covid 19?	No	639	81.8	302	84.1	355	74.7	1296	80.2	0.001
	Yes	143	17.3	57	15.9	120	25.3	320	19.8	
Do you fear you may contract the coronavirus?	No	721	92.2	213	59.3	193	40.6	1127	69.7	<0.001
	Yes	61	7.8	146	40.7	282	59.4	489	30.3	

**Figure 4:**
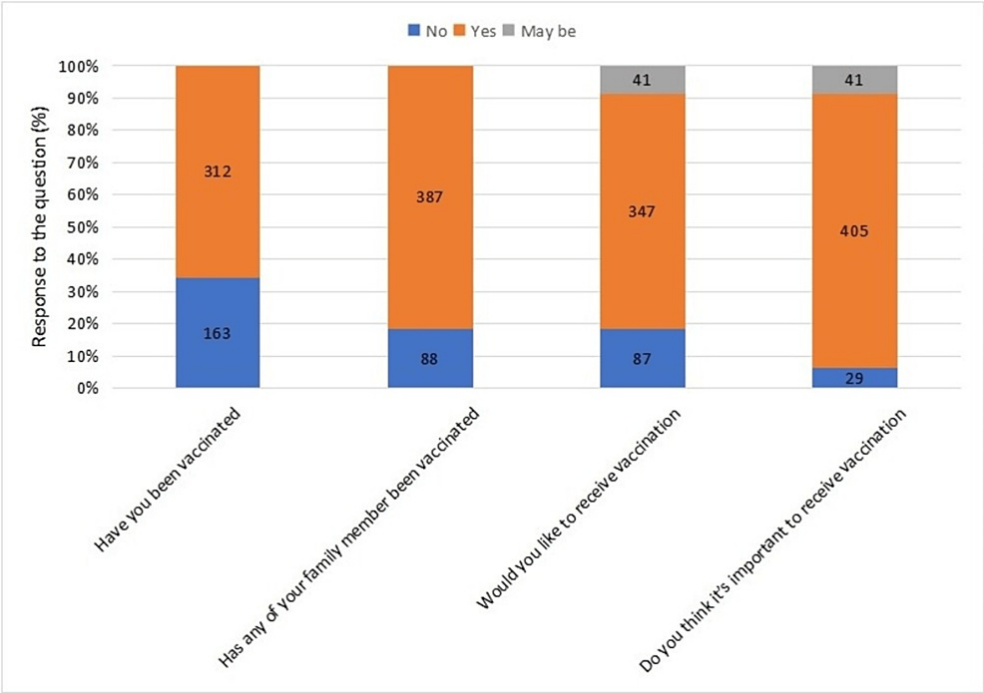
Vaccination status of respondents and their family members.

The vaccination status was only valid during the third wave of COVID-19. Figure 4 shows that 66% of the respondents were vaccinated, and more than 80% had their eligible family members vaccinated. Among them, 347 (73.1%) showed a willingness to receive vaccination, and 405 (85.3%) considered it important to receive vaccination. In this study, binary logistic regression analysis was performed to determine the factors associated with COVID-19 testing through the three major waves in Punjab, Pakistan (Table 5). For the association between testing and FLS, the adjusted odds ratio was 1.62 (95% CI: 1.21-2.16), which means that the odds of being tested for COVID-19 were 1.62 times higher for individuals with flu-like symptoms compared to those without these symptoms. Among the age groups, 31-40 (2.00; 95% CI: 1.13 - 3.53) years was significantly associated with higher testing rates. This means that the odds of being tested were 2 times higher for individuals aged 31-40 years in contrast to other age groups. History of traveling abroad was also significantly associated with higher testing rates, with an adjusted odds ratio of 3.08 (95% CI: 1.50-6.33). This means that the odds of being tested were 3.08 times higher for individuals with a history of traveling abroad compared to those without such a history. In contrast, other age groups, gender, and inland traveling were not significantly associated with testing. Finally, another factor associated with testing with COVID-19 waves, the odds of being tested increased dramatically from the first to the third wave. Compared to the first wave, the odds of being tested were 3.27 times higher during the second wave (3.27; 95% CI: 2.09 - 5.12) and 13.75 times higher during the third wave (13.75; 95% CI: 9.43) (Table 5).

**Table 5:** Binary logistic regression model for association of COVID-19 testing with respect to waves, taking FLS, age, gender, and traveling history. FLS: Flu-like symptoms

		Tested for Coronavirus?				Adjusted odds Ratio (95% CI)
		No		Yes		
		n	%	n	%	
Flu-like symptoms	No	655	83.2	132	16.8	Ref
	Yes	658	79.4	171	20.6	1.65 (1.24 – 2.19)
Time	First wave	739	94.5	43	5.5	Ref
	Second wave	304	84.7	55	15.3	3.27 (2.09 – 5.12)
	Third wave	270	56.8	205	43.2	13.75 (9.43 – 20.04)
Age	10 - 20	335	83.5	66	16.5	Ref
	21 - 30	800	81.3	184	18.7	1.15 (0.81 – 1.63)
	31 - 40	94	77.0	28	23.0	2.00 (1.13 – 3.53)
	41 - 50	35	68.6	16	31.4	1.33 (0.65 – 2.72)
	> 50	49	84.5	9	15.5	0.66 (0.07 – 6.36)
Gender:	Male	402	81.7	90	18.3	Ref
	Female	911	81.0	213	19.0	1.10 (0.81 – 1.50)
Traveled abroad	No	1287	81.8	286	18.2	Ref
	Yes	26	60.5	17	39.5	3.08 (1.50 – 6.33)
Traveled inland	No	857	85.5	145	14.5	Ref
	Yes	456	74.3	158	25.7	0.82 – 1.48)

## Discussion

Despite being the world's fifth-most-populous country, Pakistan has recorded the world's 29th-highest death toll (at approximately 23,087) and 29th-highest number of confirmed cases (at approximately 1,011,708 much lower than its neighbors India and Iran till 2021) [11,12]. Pakistan was the first country to suggest and implement a smart lockdown strategy to combat one of the worst pandemics in history [13]. On the contrary, unfortunately, Pakistan also ranks 103rd out of 132 countries in testing capacity [14]. Pakistan’s lower COVID-19 infection rate is often attributed to both the smart lockdown strategy and the low testing capacity. However, this study was conducted to determine whether people’s attitude towards testing was also a significant factor in the under-testing and under-reporting of COVID-19 in Pakistan. To the best of our knowledge, this is the first study in Pakistan designed to determine the COVID-19 testing trends in association with FLS, fear, and traveling in all three major waves in Pakistan.

Among 1616 participants, the age group that engaged the most (60.9%) in all three waves was from 21 to 30 years with almost two-thirds being females (69.6%). There is a gradual progressive increase in inland and outland traveling with each wave which is indicative of Pakistan’s national policy of smart lockdown across the country and on and off opening of land, sea, and air borders with other countries for the purpose of trade to relieve economic burdens. The smart lockdown strategy and porous borders both are strongly suggestive of higher infection spread within the country [11,15].

However, overall, in all three waves, only 18.8% (less than one-third) of the participants reported COVID-19 testing with 51.3% (almost half) reporting to have had FLS as well and 35.6% (slightly over one-third) also reported being exposed to FLS by their families (Table 4). The testing rate in this study is quite similar to another study conducted in Pakistan which reported a testing rate of 16.8% [13]. This strongly suggests that the overall testing trend remained low despite high rates of symptoms and exposure which strongly suggests under-reporting of the COVID-19 cases in Pakistan. This observation is also strongly aligned with the national study conducted by Aheron et al. (under the supervision of the US Centre for Disease Control and Prevention) who modeled suspected and confirmed COVID-19 cases to be 3 times as many suspected and confirmed COVID-19 cases than reported [13]. However with time, encouragingly, the testing trend increased (first wave = 5.5%, second wave = 15.3%, third wave = 43.2%) as exposure also increased with the country relaxing its social distancing restrictions due to pressing economic pressures [11,14].

A crucial factor to consider and draw attention to is the fear and stigma attached to COVID-19 and hospitalization [15]. As evident in the literature, fear and stigma around COVID-19 were higher in the earlier phases of the pandemic [16,17]. This raises some pertinent questions here; first, could fear and stigma be a discouraging factor and counterproductive in the uptake of COVID-19 testing and thus under-reporting of it as suggestive of in our study as well? In this study, although fear of contracting COVID-19 was quite high (69.7%) it was not an encouraging factor for testing among the Pakistani population. Second, with time more information on COVID-19, its management, and prevention started to become available reducing uncertainty and fear, could that have positively affected the perception and attitude of people towards COVID-19 testing? Thirdly, did the government’s response and policy on COVID-19 impact have any role in creating the illusion of fear and stigma which in turn affected the behavior of people toward COVID-19 testing? These questions may be extremely useful in understanding the barriers to the uptake of infectious disease testing and consequently disease management and prevention. Therefore, it is suggested that further detailed studies be conducted to explore the association of fear with the uptake of COVID-19 testing.

International traveling for education and employment unlike fear was rather very strongly positively associated with testing which shows that legal requirements were a positive factor in the increased testing trend which again is an observation made by Aheron et al. as well. Hence it may not be unsafe to say that measures and interventions oriented around education and employment sectors may be effective ways to modify people’s behavior toward infectious disease prevention and management [13].

The COVID-19 positivity rate within the first wave was 39.5% for the participants and 17.3% for their family members, closely aligning with a study by Imran et al. (2021) from Pakistan, which reported rates between 18% and 23%. In the second wave, the positivity rates were slightly elevated at 35.1% for participants and 15.9% for family members (compared to 8% and 11%), while the third wave was not investigated. These numbers again suggest that despite a relatively higher infection rate which means that the chances of spread were higher, only 18.8% of the people opted for COVID-19, which reaffirms the argument of under-testing and under-reporting of COVID-19 [18].

The study was limited by the fact that data was collected through online surveys, which may have limited the representation of certain population sub-groups, thus affecting the generalizability of the study. Also, the FLS and exposure to FLS are self-reported; hence, there is a risk of recall or reporting bias.

## Conclusions

In conclusion, it is not unsafe to say that in Pakistan COVID-19 has been relatively under-tested and under-reported as compared to its prevalence and exposure. Reasons why low uptake of COVID-19 testing was a trend among the Pakistani population despite free/subsidized testing policy need to be further investigated. This study informs the policymakers, government, and healthcare professionals that fear of an infectious disease may not significantly change the behavior of an economically challenged lower middle-income country (LMIC) population like Pakistan, but legal, economic, and educational needs may be efficiently utilized to do so. It further informs that any future policy/ intervention that puts an economic load on the people may not be well adhered to in LMICs. This observation in the study strongly implies that any one policy/intervention (such as country-wide lockdowns) for an infectious disease may not be an effective intervention across the globe and should be customized based on the population’s needs and challenges. Pakistan did so by improvising the social distancing policy from country-wide lockdowns to smart lockdowns and it worked well for the country too. However, that was a decision made under immense pressure in a challenging, unpredictable situation with the entire economic and healthcare systems at stake. Thus, it is incumbent upon LMICs to conduct more research around infectious disease prevention and management in line with the established guidelines yet more customized to their context when stakes are low to better prepare for any such future disaster. More preparedness may generate a more confident, reliable response from the government in case of infectious disease spread which may be a crucial factor in gaining the trust of people, and hence more acceptability of infectious disease testing, management, and vaccinations that Pakistan already struggles with in case of polio and hepatitis-B.
